# Clinician and researcher responses to the term pain catastrophizing and whether new terminology is needed: Content analysis of international, cross-sectional, qualitative survey data

**DOI:** 10.1016/j.jpain.2025.105330

**Published:** 2025-02-05

**Authors:** Hannah Boyd, Dokyoung S. You, Angela Nguyen, Laura Connoy, Devdeep Ahuja, Christine Chambers, Penny Cowan, Rachel Cox, Geert Crombez, Amanda B. Feinstein, Anne Fuqua, Gadi Gilam, Sean C. Mackey, Lance M. McCracken, Lynn M. Martire, Kathleen Sluka, Peter O’Sullivan, Judith A. Turner, Christin Veasley, Maisa S. Ziadni, Claire E. Ashton-James, Fiona Webster, Beth D. Darnall

**Affiliations:** a Stanford Pain Relief Innovations Lab, Division of Pain Medicine, Department of Anesthesilogy, Perioperative and Pain Medicine, Stanford University, Palo Alto, CA United States; b Department of Psychology, Uppsala University, Uppsala, Sweden; c Division of Pain Medicine, Department of Anesthesiology, Perioperative and Pain Medicine, Stanford University, Palo Alto, CA, United States; d Stanford University, 450 Jane Stanford Way, Stanford, CA, United States; e Arthur Labatt Family School of Nursing, Western University, London, ON, Canada; f RTW Plus, Tintagel House, 92 Embankment, London SE1 7TY, United Kingdom; g Departments of Psychology & Neuroscience and Pediatrics, Dalhousie University and Centre for Pediatric Pain Research, IWK Health, Nova Scotia, Canada; h World Patients Alliance, Washington, DC, United States; i Department of Experimental-Clinical and Health Psychology, Ghent University, Belgium; j Department of Anesthesiology, Children’s Healthcare of Atlanta, Atlanta, GA, United States; k University of Alabama-Birmingham, Birmingham, AL, United States; l The Institute of Biomedical and Oral Research, Faculty of Dental Medicine, Hebrew University of Jerusalem, Jerusalem 91120, Israel; m Department of Human Development and Family Studies, The Pennsylvania State University, PA, United States; n Department of Physical Therapy and Rehabilitation Science, The University of Iowa Carver College of Medicine, IA, United States; o School of Allied Health, Curtin University, Western Australia, Australia; p Department of Psychiatry & Behavioral Sciences and Department of Rehabilitation Medicine, University of Washington School of Medicine, Seattle, WA, United States; q Chronic Pain Research Alliance, Milwaukee, WI, United States; r Pain Management Research Institute, Kolling Institute, Sydney Medical School, Faculty of Medicine and Health, The University of Sydney, Australia

**Keywords:** Chronic pain, Pain catastrophizing, Stigma, Patient-centered communication, Qualitative

## Abstract

Pain catastrophizing is understood as a negative cognitive and emotional response to pain. Researchers, clinicians, advocates, and patients have reported stigmatizing effects of the term on patients when used clinically and in the media. This report describes the results of an international, observational, cross-sectional study investigation of clinician and researcher (professionals) perspectives on the term pain catastrophizing and whether new terminology is needed or desired. Open-ended electronic surveys were distributed to researchers and clinicians by collaborators, stakeholders, and through social media. Professionals reported on their familiarity with the term, its meaning and impacts, and their use of the term with patients. 1397 surveys from professionals in 46 countries (48.5% from the U.S.) were received. The sample was almost two-thirds female (61.3%), with a mean age of 56.67 (SD=4.04) years, and comprised of 78.6% clinicians (63.6%, pain specialists; n=698) and 20.3% researchers. The majority were familiar with the term (82.2%; n=1148). Among the 1098 clinicians, 33.6% had used the term in communication with patients. A content analysis of professionals’ responses to open-ended questions is presented. Coded responses were synthesized into five content categories or themes: (1) pain catastrophizing is an exaggerated response to pain; (2) pain catastrophizing is an unhelpful response to pain; (3) the term pain catastrophizing is stigmatizing; (4) the term pain catastrophizing is clinically useful; (5) patients’ perception of the term varies. Results highlight the continual controversy surrounding the term pain catastrophizing and the need for additional research and education to incorporate patient-centered approaches into clinical and public communications.

**Perspective:**

We present a content analysis of international clinician and researcher perspectives on the term pain catastrophizing. This investigation provides the largest depiction to date of the controversy surrounding pain catastrophizing and may guide future efforts to decrease stigma in patients with chronic pain and improve patient-clinician communication.

## 1. Introduction

Clinician communication influences patient outcomes in pain management. The words that clinicians use to explain pain and treatment approaches can influence treatment outcomes by altering patient perceptions of clinician trustworthiness, treatment expectations, and treatment adherence^1,2^ and outcomes.^3–6^ Further, patients are more likely to follow treatment protocols when they feel understood by their clinicians and cared for.^7,8^

A patient-centered approach supports the delivery of empathetic, effective care and rapport building, which results in more positive clinical encounters for both patients and clinicians.^9^ Positive, patient-centered language is a crucial element of patient-centered care. Stigmatizing and deprecating language contributes to negative patient experiences, distress, and reduced perceived quality of care.^10–12^ Hence, it is essential that we take steps to identify such language used by clinicians.

Pain catastrophizing is a term that has generated controversy within pain care, academic articles, media stories, and other public communications; a criticism of the term is that it is stigmatizing to patients.^13–16^

The term “catastrophizing” was first coined by Albert Ellis in 1962 within the context of cognitive psychology.^17^ Ellis defined the term as “exaggerating adversities into something far worse than they actually are; seeing things at their worst when they are sometimes relatively minor; and greatly exaggerating the frequency and/or danger of something you dislike.”^18^ Later, Aaron Beck applied the term to describe maladaptive cognitions in those with anxiety and depression.^19,20^ In 1983, Rosentiel and Keefe created the pain catastrophizing subscale of the Coping Strategy Questionnaire to measure pain-related cognition and coping.^21^ In 1995, Sullivan et al. published a 13-item measure of pain catastrophizing, the Pain Catastrophizing Scale (PCS), which includes subscales for rumination, magnification, and helplessness.^22^ More than 30 years of accumulated evidence substantiates the direct association of pain catastrophizing with worse patient outcomes for pain intensity,^23–26^ depression,^27–29^ disability,^30–33^ surgery,^26,34^ pain treatment,^27,35^ spouse responses,^12,36^ and quality of life.^35,37–39^ Research documents substantial outcomes and benefits for treatments that reduce pain catastrophizing scores.^40–43^

**Fig. 1.**
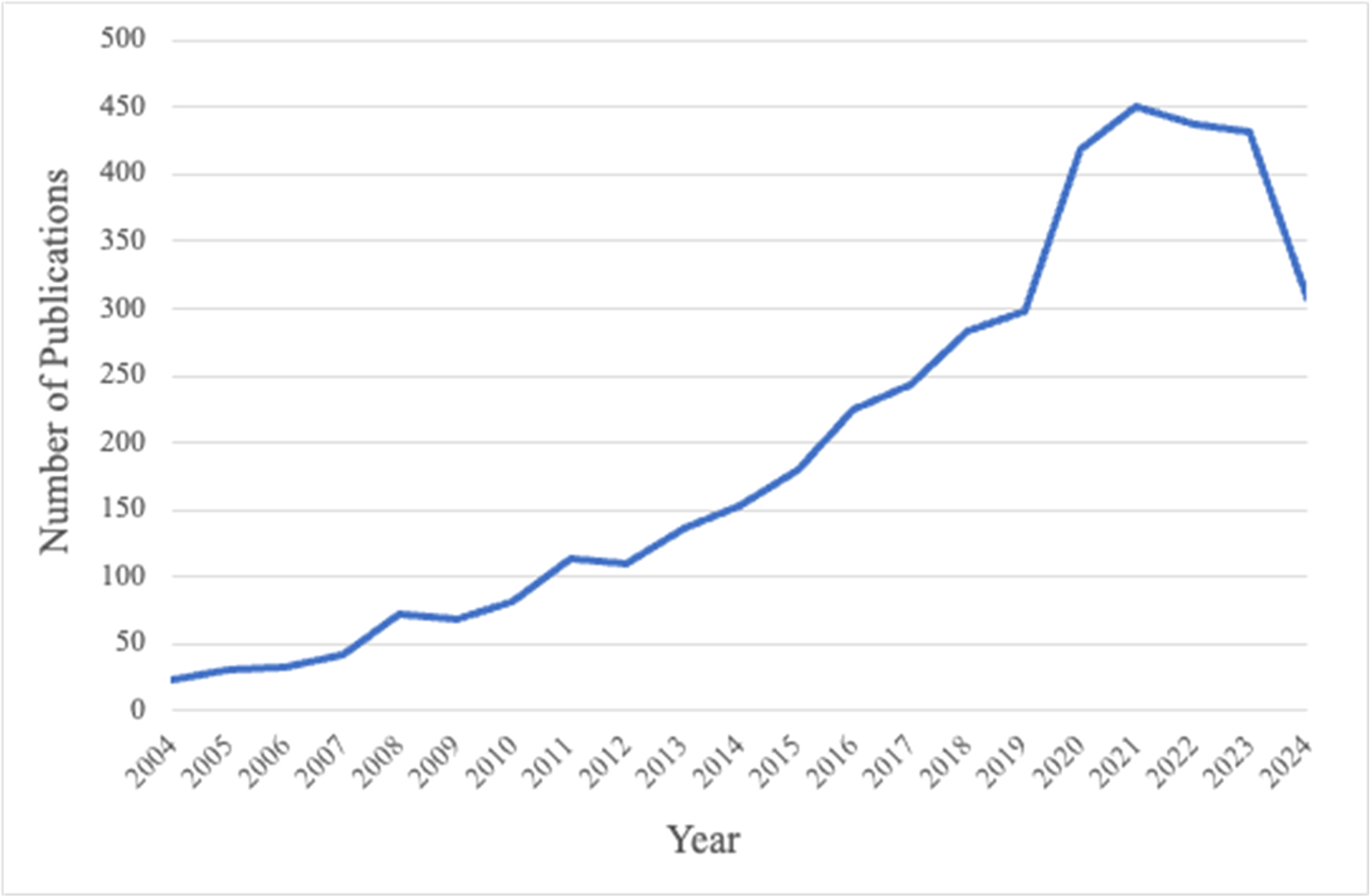
Number of PubMed publications by year containing pain catastrophizing as a keyword.

A 2024 PubMed search identified 3380 scientific articles that used pain catastrophizing as a keyword, with Fig. 1 depicting the annual upward trajectory of the term’s use, with more than 2000 articles published since 2018. Increased scientific publications foster greater use of the term in conference presentations, clinical care, and in media stories read by the public.^17,41,44–46^

A broader investigation of the term pain catastrophizing involved two components: patient perspectives, with the 2023 published results briefly described below, and clinician and researcher perspectives, the subject of the current report. The published analysis of patient perspectives included responses from 3521 patients and caregivers residing in 46 countries.^22^

About 45% reported having heard of the term pain catastrophizing; 12% (n= 349) reported having been described as a “pain catastrophizer” by a clinician. 32% percent perceived the term as problematic and/or stigmatizing. The purpose of the patient data was not to arrive at consensus or majority perspective; rather, the goal was to elucidate the range of patient perceptions to better appreciate any stigmatizing effects.

The current report describes results for the clinicians and researcher survey that was advertised and administered internationally in concert with the patient survey described above.^13^ Professionals were invited to provide their responses and perspectives in a survey that contained both quantitative and open-ended questions on the term pain catastrophizing, with a stated project goal of understanding whether new terminology was needed.

The aims of the current study were to explore clinician and researcher: (1) familiarity with the term pain catastrophizing; (2) conceptualizations of the construct; (3) perspectives regarding how the term pain catastrophizing impacts patients; and (4) perspectives on whether an alternate term might be needed or useful.

## 2. Methods

This patient-centered investigation was generated from unsolicited and direct patient requests, received in 2019, to undertake such a study. The study team included 12 patient stakeholders who helped design the study, including generating the study surveys that were distributed to patients (the prior study) and to clinicians and researchers (the current study). Patient stakeholders helped design our study website and distribute our surveys. Patient stakeholders have been involved in the manuscript writing process and included as authors.

The study design was an observational, cross-sectional, single time point survey administration with broad and international distribution of study invitations. The Stanford University Institutional Review Board determined exempt status for the study and thus informed consent was not applicable. All study advertisements were electronic and included brief introductory language regarding an unpaid and anonymous opinion survey on the term pain catastrophizing. The study advertisements contained a link to the Stanford study website where the following introductory language was displayed:

“This is a patient-centered project being led by a group of committed pain researchers, patients, patient advocates, and healthcare professionals. We aim to understand the perspective of patients, researchers and healthcare professionals with regard to the term ‘pain catastrophizing.’ We will be collecting and collating the information we receive from your responses to help us understand whether it’s time for a change in the use of this term—and to possibly create new terminology that is compassionate, patient-centered, and more considerate for use in the medical community.”

Study ads and online survey invitations were distributed internationally through social media (e.g., Twitter, Facebook) and website postings, and via email distribution lists of pain organizations. The study advertisement and survey link were distributed through the social media accounts (Facebook, Instagram, Twitter) of the Stanford Division of Pain Medicine and professional and research listservs. Study investigators also emailed study information to the directors of national and international patient organizations with a request that they consider distributing the survey amongst their membership and across their social media platforms. Twelve patient stakeholder collaborators distributed the study advertisement to peers and colleagues in 4 countries. Thirty-two scientific stakeholder collaborators in 9 countries distributed the study advertisement to colleagues, relevant listservs, and patients in their respective countries. Seven national organizations (Chronic Pain Research Alliance, the American Chronic Pain Association, PainAustralia, Center for Pediatric Pain Research, Solutions for Kids in Pain Network, American Society of Anesthesiologists, Pain USA) distributed the study advertisement. Finally, 2 international organizations (Global Alliance of Partners for Pain Advocacy, an independent organization with a relationship to the International Association for the Study of Pain, and the World Patients Alliance) distributed the study advertisement among their members and via social media channels.^13^

After clicking on the study link, interested individuals were invited to choose either the clinician/researcher survey or the patient/caregiver survey. After selection of the clinician/researcher survey, participants viewed the following text (see Supplemental Professional Survey):

“Our Goal: With this survey, we aim to understand healthcare clinician and pain researcher perspectives on the term “pain catastrophizing.” This research is motivated by reports from patients and clinicians alike that the term ‘pain catastrophizing’ is unhelpful and may contribute to patient alienation and distress. As such, we are distributing a patient survey internationally to learn about the patient perspective. Our larger goal in this patient-centered work is to explore new terminology that is broadly acceptable to all stakeholders, and promotes good patient engagement and collaboration with healthcare clinicians to achieve best outcomes for their pain treatment.”

English and Spanish language versions of the professional survey were available upon selection.

Participants were asked demographic items related to age, gender, country of residence, and profession type (clinician or researcher); and, for clinicians: duration of clinical experience, clinical discipline, and whether they identified as a pain specialist or not. The survey did not assess level of pain training. Next, participants were presented with questions that assessed their knowledge and understanding of the term ‘pain catastrophizing.’ Clinicians received an additional set of questions as outlined below.

All participants were asked:

“Have you heard of the term pain catastrophizing?”

(yes/no)

Those who answered yes were then asked, “Please briefly describe what it [pain catastrophizing] means to you. If you are unsure, please just jot down a few words that come to mind that you associate with the term.”

(open-ended responses)

For clinicians only:

“Have you ever used the term ‘pain catastrophizing’ in the context of your communications with patients?”

(yes/no)

“If yes, can you describe how patients respond to the term?” (free text responses) and

“How frequently do your patients react negatively to your use of the term pain catastrophizing?”

(7-point Likert scale ranging from “Never” to “Every time”)

All participants were asked,

“Please rate how important you feel it is to create a new term for pain catastrophizing, one that would be acceptable to most patients, providers, and researchers?”

(7-item Likert scale from “Not at all important” to “Greatest Importance”)

Next, all participants received the following text and definition of pain catastrophizing:

“This is the definition of pain catastrophizing: Pain catastrophizing refers to how we respond to pain we have right now, or to pain we expect to have in the future. It includes thoughts we may have about pain (e.g., “I can’t stop thinking about how much it hurts.”), feelings about pain (such as helplessness) and expectations for future pain (e. g., “I worry that my pain will only get worse.”).

While the degree of pain catastrophizing and level of pain intensity we experience are related, research shows that they are different. We can control pain intensity in research studies (by keeping it constant) and see that pain catastrophizing – our level of pain-specific distress – changes how pain is processed in the central nervous system.”

Following the definition, participants were asked three additional questions.

“Please tell us what first comes to your mind when you hear the term pain catastrophizing.”

(free text responses)

“Please feel free to add any further comments or thoughts about what would be a better term for pain catastrophizing? There are no right or wrong answers, or bad ideas.”

(free text responses)

“Is there anything else you would like to tell us on this topic? Please feel free to attach a separate page if you’d like to provide further comments or insights.”

(free text responses)

The study survey went live online on May 29, 2020 and closed on August 17, 2020.

## 3. Data analysis

Data analysis utilized descriptive statistics and qualitative methods for open-ended responses and content analysis. Demographic items, Likert scale responses, and items with binary response sets were summarized as frequencies (Table 1). Five open-ended questions (Table 2) were analyzed using content analysis.^44,47–49^ We used the following definition of content analysis as a *“research technique for making replicable and valid inferences from texts (or other meaningful matter) to the contexts of their use.”*^50^.

Content analysis relies on coding and categorizing of data. Coding can be either a priori or emergent. With emergent coding, codes are independently reviewed; the reviewers meet, compare notes and develop a coding framework which is applied to the rest of the data. We maintained research rigor through enhancing credibility^51^ by following this process of developing codes and content categories or themes collaboratively, and the inclusion of rich, expansive and verbatim participant responses for the reader to judge the representativeness or fit between quotes and themes.^52^ Through extensive discussion between coders, (HB, SY, AY) we made subsequent iterations of the framework for the clinician and researcher data. We also supplied counts/-frequencies for the reported themes and sub-themes. Although reporting such data is not consistent with research designs that are qualitative from their inception, we included it as relevant for large survey data and for readers curious about the prevalence of each theme and sub-theme. Where applicable, we also included frequencies for each code within themes to give readers a better understanding of the robustness of each theme.

As an initial step, SY and HB independently read the first 25 responses and identified codes; they then met to discuss their codes. This was not done to achieve consensus but rather as a standard way of achieving reflexivity through sharing and understanding differing interpretations of the same data.^48^ Two student coders, RC and AY, then coded the same 25 responses, repeating the process of comparing and discussing codes. FW, HB, SY, LC, and BD then met to collaboratively create the final coding framework that was applied across the remaining data set by the 4 coders. Throughout this process, the four coders met periodically to discuss and confirm their evolving interpretations of the data, challenge assumptions and deepen their understanding of the data. Conceptually-related codes were then grouped to identify meaningful patterns (themes) based on discussions among initial coding creators, stakeholders, and methodological experts (FW, HB, SY, LC, CAJ, and BD).

As a separate process, frequencies (n values) were reported for both demographic items and individual codes and represent the number of participants included within the category. Frequencies for themes and sub-themes reported the number of total responses coded under that theme, as individuals may have provided responses that fell under multiple codes included in a theme.

## 4. Results

A total of 1690 self-identified clinicians and researchers initiated the professional survey. Participants who provided demographic information only (i.e., no qualitative data; n=286) were excluded, as were participants who were identified to be neither clinicians nor researchers (n=7). Thus, the final thematic analytic sample included n=1397 clinicians and researchers.

### 4.1. Countries represented in the study sample

The 1397 participants resided in 46 countries, with the largest proportion of participants from the United States (n=677, 48.5%), then Canada (n=145, 10.4%), Australia (n=121, 8.7%), the United Kingdom (n=111, 7.9%), India (n=102, 7.3%), Singapore (n=54, 3.9%), and Portugal (n=22, 1.6%). Ireland (n=13), Brazil (n=13), Belgium (n=12), and New Zealand (n=11) each represented less than 1% of participants. The Other category (n=116, 8.3%) was comprised of participants from Indonesia, Afghanistan, Pakistan, South Africa, Nepal, the Netherlands, United Arab Emirates, Jordan, Sweden, Japan, Egypt, France, Sri Lanka, Spain, Mexico, Ecuador, Germany, Norway, Colombia, Italy, Denmark, Israel, Switzerland, Saudi Arabia, Malaysia, Chile, Argentina, Angola, Iran, Luxembourg, Hong Kong, Latvia, Venezuela, and Poland.

### 4.2. Other demographics and familiarity with the term and use of the term

Other participant characteristics are presented in Table 1. The sample was predominantly clinicians (78.3%), 61.4% were female (61.4%), and the mean age was 56.67 years (SD 4.04). Over half of the clinician participants reported being pain specialists (63.5%) and being in clinical practice for more than 10 years (58.3%).

The majority of the sample was familiar with the term pain catastrophizing, with 82.2% of the full sample (n=1148) reporting yes and 16.9% (n=235) reporting no when asked, “Have you heard of the term pain catastrophizing?” 1.0% did not respond (n=14). Furthermore, among the 1098 clinicians, 33.6% (n=369) reported using the term pain catastrophizing in the context of communication with their patients, and thus had clinical experience with implementation of the term and understanding patient responses to their use of the term. Contextual data is captured below in the thematic analyses of the open-ended survey items.

### 4.3. Content analysis and identified themes

1397 clinicians and researchers provided text responses to the free text questions. Table 2 displays the free text questions and the number of participants (n) who responded to each question. The study team created codes to categorize responses and to allow description of the magnitude of participant responses within each code. These codes were synthesized into conceptually-related categories resulting in the five content areas or themes presented in this report. The counts for responses used in the content analysis can exceed the sample size because multiple free text responses were combined across items. Below, we report identified themes and general frequencies for responses that align with each theme along with example quotes. We report data by individual participants for codes that fall within each theme, but not for themes themselves.

In analyzing all combined free text responses, 5 primary content themes were identified: (1) pain catastrophizing is an exaggerated response to pain; (2) pain catastrophizing is an unhelpful response to pain; (3) the term pain catastrophizing is stigmatizing; (4) the term catastrophizing is clinically useful; (5) patient’s perception of the term vary. Finally, results describe clinicians’ suggestions for a replacement term.

### 4.4. Theme 1: pain catastrophizing is an exaggerated response to pain (n=2056 responses)

Many responses accurately captured the definition of pain catastrophizing as a heightened pain response (n=876) involving amplified negative cognitive and emotional components (n=1080). Some of these responses were captured as exaggeration (231 participants) or magnification/amplification (258 participants), concepts that are rooted in the clinical definition of the term pain catastrophizing.

However, other responses extended into descriptions that were more extreme than the definition, such as describing the construct as an overreaction (610 participants), dramatization (56 participants), faking (45 participants), and hysterical behavior (13 participants). There was a general sentiment that among patients with a similar condition, those engaging in pain catastrophizing exhibited higher levels of pain responses and that these responses were interpreted as being out of proportion or unjustified.

“That the person is over-exaggerating their pain and that this then increases their pain experience.”

(Participant #610)

“In most clinical practice settings this term is used to most often describe a patient who is exaggerating physical pain they feel or suffering from severe anxiety regarding pain which is not nearly as bad as they actually feel.”

(Participant #974)

Some participants (n=400) defined pain catastrophizing by comparing patients who catastrophized to “the average person”:

“tendency to describe a pain experience in more exaggerated terms than the average person, to ruminate on it more and/or to feel more helpless about the experience.

Seeing the cup as ‘half-empty’ instead of ‘half-full’.”

(Participant #395)

“excessive rumination over a painful condition that typically would not provoke this response in most people.”

(Participant #1535, emphasis ours)

However, there was an acknowledgement that healthcare professionals’ interpretation of these exaggerated behaviors may be inaccurate or wrongly used.

“It has negative connotations that we don’t believe their pain is that bad - that the patients are hysterical and exaggerating the pain levels and symptoms.”

(Participant #157).

“We used to give out questionnaires pre and post attendance and one was called the pain catastrophising scale. It was labelled that on the paper. I hated it because it basically said ‘you’re just being a drama queen.’”

(Participant #1280)

Healthcare professionals also related exaggerated pain responses to psychological processes including worry/anxiety (n=193 participants), fear (n=135 participants), rumination (n=263 participants), helplessness (n=93 participants), and hopelessness (n=51 participants).

For example,

“In most clinical practice settings this term is used to most often describe a patient who is exaggerating physical pain they feel or suffering from severe anxiety regarding pain which is not nearly as bad as they actually feel.”

(Participant #974)

“Inappropriately cognitive response to pain that involves negative thinking regarding the pain experience.”

(Participant #1461)

A small number of responses associated pain catastrophizing with mental illness (n=44 participants). Responses were coded as general psychological/emotional component (n=401 participants) when they broadly discussed thoughts and emotions as being central to pain catastrophizing.

“…psychological phenomenon describing a patient’s illness narrative of their pain and illness. A way of measuring the level to which experience of pain is interpreted in a negative and cataclysmic way by patients.”

(Participant #387)

irrational or exaggerated beliefs related to the meaning of pain, future outcomes, and anticipation of pain

(Participant #486)

### 4.5. Theme 2: pain catastrophizing is an unhelpful response to pain (n=610)

Healthcare professionals also expressed the idea that the consequences of pain catastrophizing are unhelpful, leading to increasing pain, a diminished ability to function, and preventing improvement in response to treatment interventions. They expressed the concept as a maladaptive cycle (n=308) in which pain catastrophizing itself increases the negative thoughts and emotions within a patient’s experiences.

“When the fear of pain becomes one’s primary focus driving neurological system into a higher level of sensitivity, leading to more pain.”

(Participant #1367)

“A response to painful stimuli that includes behavioral and cognitive factors including ruminating, avoidance, and other distress that tend to exacerbate the painful experience.”

(Participant #1313)

“Disordered response to ongoing pain effecting change in affect, promoting avoidance such as kinesiophobia and numerous behavioral issues often limiting patient’s willingness to and response to treatment.”

(Participant #206)

“Someone who catastrophizes is likely to experience more anxiety and stress associated with the experience of pain due to a focus on the negative aspects of their lived experience and what it may mean for them in the future.”

(Participant #398)

“When a person worries about the future negative effects of the pain they are experiencing currently and the worries can contribute to their current distress and may even slow their recovery.”

(Participant #68)

The notion that pain catastrophizing was related to the patient’s difficulty coping with their situation was also coded (n=393 participants). For example, the following references:

“For me, it seems to be apparent when a patient is explicitly overwhelmed and overly focused on their pain in such a way that is unhelpful or detracting from active coping or in taking actionable steps to improve their condition or life.”

(Participant #167)

“People are finding their pain experience overwhelming to manage and as a barrier to doing what matters most to them.”

(Participant #258)

A subgroup of participants (n=39) expressed that patient pain experiences defined as pain catastrophizing are actually normal responses to pain. Examples include:

“the term needs to reflect that it is normal to have distress or fear, and normal for this distress to fluctuate.”

(Participant #1684)

“The tendency to worry about pain and worry about potential consequences is a normal part of the pain experience and should be met with empathy and reassurance.”

(Participant #456)

### 4.6. Theme 3: the term pain catastrophizing is stigmatizing (n=482)

Participants described the stigmatizing nature of the term, voicing general issues with how other healthcare professionals used the term and identifying its negative impact on patients. More specifically, they associated it with the following five codes: misuse by the healthcare system (50 participants), blaming (59 participants), dismissive/invalidating (171 participants), negative/inaccurate term (146 participants), and general judgment or negative impact (60 participants).

In the following quoted responses, the overall judgmental nature of the term pain catastrophizing was denoted:

“ITS INNAPROPRIATE AND UNETHICAL. These are not descriptive terms that a physician or any other practitioner should be using to describe a patient. I think the term should be put in a sling/urban dictionary for historical context as a derogatory term used against patients who are suffering, by unethical caregivers.”

(Participant #162)

“A judgment made by someone other than a patient that the patient’s response (usually chronic) to the pain they are reporting is excessive for what they are experiencing. AND Professionals and parents who make judgements without being fully empathic towards the patient.”

(Participant #1684)

One participant compared pain catastrophizing to the stigmatizing effect of hysteria as a gendered term.

“It reminds me of the term ‘histrionic’ that is used against women in a demeaning manner to explain her emotions.”

(Participant #200)

Other participants referenced known facets of stigmatization, namely, invalidation, blame, and dismissal and discrimination.

“Pejorative minimization and invalidation of a person’s experience of chronic pain.”

(Participant #550)

“The use of Pain catastrophizing in most usage I have heard means they really are not hurting and throw them out.; It is used in a negative manner to rationalize lack of treatment and dismiss pain patients who are in need of help.”

(Participant #877)

“Yes, some do exaggerate, but now it has become the norm to basically call all pt with chronic pain/debilitating pain liars. Then they can justify not treating the pt, or not treating them appropriately. It is used as a justification to not only allow pt to suffer needlessly, but to also mentally abuse these people.”

(Participant #171)

“It’s a patronizing term. I would not use this term with a patient. No one tells a patient with chronic asthma that they are “asthma catastrophizing.” I am concerned that many pain patients are not getting the medical treatment they need for their pain because they are being told the pain is in their head. While I don’t specialize in treating pain, I do specialize in cognitive behavioral therapy, and I understand its strengths and limitations. Patients with pain need to feel empathy and compassion from their clinicians, and words like “catastrophizing” are not empathetic.”

(Participant #188)

“We as doctors have created this term to allow us to save face in the context of no explanation for someone’s suffering- it blames the patient.”

(Participant #1277)

Moreover, 55 clinicians explicitly reported not using the term with patients because they found it inappropriate or ineffective. For example,

I try not to use it because I consider it derogatory.

(Participant #1155)

A small subset (n=8) of healthcare professionals had personal experience with chronic pain and identified with the patient’s experience of stigmatization.

“It means feeling that every small setback will cascade into a series of negatively escalating events. I want to say I am also the mother of a [child] very with severe chronic pain and have done it myself.”

(Participant #155)

“An UNWARRANTED, OVER REACTION by the person experiencing pain, which is not or cannot be felt or understood by the healthcare professional. Pain is multifaceted and term feels judge mental and limits understanding of the persons pain experience, as someone who has been living with chronic pain for many years.”

(Participant #274)

### 4.7. Theme 4: the term pain catastrophizing is clinically useful (n=182 responses)

The fourth theme captured the variability in perceptions of the term’s utility. While many clinicians and researchers believed the term pain catastrophizing should be changed, as seen in Theme 3, professionals believed it was useful in some contexts. 47 participants actively used the term and questioned the need for changing it.

“The term “pain catastrophizing” is a good one, with a good body of research associated with it. I would suggest to keep it.”

(Participant #471)

“I think the word catastrophizing is a great word because patients need to be aware of the significance of their pain perception. Using a gentler word will take meaning away from the term. I will continue to use catastrophizing and think this is not a good idea [to change it].”

(Participant #1248)

Other healthcare professionals and researchers saw both negatives and positives of the term (n=35 participants). They noted the term’s accuracy, but also recognized issues when used with patients, differentiating between the term’s efficacy in research versus clinical practice.

“Although the term Pain Catastrophising may be alarming when used to describe clinical presentation of pain it perfectly describes the issue.”

(Participant #24)

“But it is important that the term is applicable to OUR use when conversing with each other or studying this construct. It is unhelpful and likely harmful to use this term with our patients.”

(Participant #591)

Finally, 101 clinicians noted that explanation or education was necessary for patient acceptance and subsequent treatment utility.

“I prefer to explain it in simpler, more personally relevant and acceptable ways.”

(Participant #167)

“We share the term respectfully and from an educational perspective. When used with the appropriate educational approach and respectfully, it is a core concept for helping people who live with pain and their care providers to understand our response to pain.”

(Participant #552)

The response of participant #552 above also provides an important illustration of the clinician’s perspective that when used sensitively and with full explanation, the term pain catastrophizing can be an important and useful aspect of clinical care. Other clinicians noted that proper education can actually change the way patients respond to the term.

“Overall negative initially, but I take time to explain it to them and take the stigma off of the word. I tell them I don’t like the word myself and give them resources to further educate themselves.”

(Participant #77)

We share the term respectfully and from an educational perspective. When used with the appropriate educational approach and respectfully, it is a core concept for helping people who live with pain and their care providers to understand our response to pain. (Participant #552).

### 4.8. Theme 5: patient perceptions of the term vary (n=242)

Of the 369 (33.6%) clinicians who reported having used the term pain catastrophizing with patients, 312 elaborated on the response of those patients. Responses were coded broadly in terms of valence as follows: neutral (n=115 participants), negative (n=78 participants), and positive (n=48 participants). These responses highlight the tendency for individual patients to react very differently.

Negative responses are exemplified in the quotes below:

“They do not like to be categorized as a catastrophizer.”

(Participant #152)

“Often with negative connotations- ”that’s not me.”

(Participant #408)

In contrast, positive quotes included:

“They identified with it.”

(Participant #471)

“Certain patients respond positively to the recognition of catastrophizing.”

(Participant #629)

Some participants expressed that patient responses are dependent on the delivery of the term by the clinician, mirroring those responses captured in Theme 3 who expressed that education was a necessary component of the term’s utility.

“Respond well if I could use the term pain catastrophising without making it pejorative.”

(Participant #406)

“But patients seemed to agree with the term once I explained.”

(Participant #1099)

Neutral responses included clinician perceptions of patient curiosity, confusion, and understanding of the term.

For example,

“Most often patients respond with curiosity regarding these different thinking styles.”

(Participant #11)

“They gain an understanding of the term.”

(Participant #1269)

“They are usually unfamiliar with this word.”.

(Participant #887)

### 4.9. Discussion

This international cross-sectional survey study included 1397 healthcare professionals from 45 countries. While 82% were familiar with the term “pain catastrophizing,” only one-third reported using the term with patients. Respondents conveyed mixed perspectives on its usefulness and appropriateness in research and practice. Some highlighted its clinical importance (Theme 4), noting it helps direct patients toward cognitive behavioral therapy-based treatments when their thoughts or emotional and behavioral responses to pain are deemed unhelpful (Theme 2). However, many expressed concerns about using the term with or about patients, citing its potentially stigmatizing nature (Theme 3) and the risk of negative patient reactions (Theme 5).

The difficulty in interpreting respondents’ concerns or support for “pain catastrophizing” is its varied meanings, as highlighted in Theme 1 of this study. Most professionals maintained that pain catastrophizing is an abnormal process involving behaviors more extreme than those of an average individual, while others viewed it as a normal response. Some aligned with Sullivan et al.’s original definition— “an exaggerated negative mental set brought to bear during actual or anticipated pain experience,” associated with rumination and feelings of helplessness^14, 22^— whereas others equated it with faking, hysteria, or an otherwise *unjustified* response to pain. In other words, some interpret pain catastrophizing as being exaggerated but *proportional* to the degree of rumination and helplessness experienced by the individual, while others view it as exaggerated and *disproportionate to* what one (presumably the clinician) would expect under the circumstances. Given the differing interpretations of the term pain catastrophizing, it is unsurprising that respondents expressed varying views on its impact on patients (i.e., stigmatizing) and its clinical usefulness in practice.

A potential challenge with the construct of pain catastrophizing as an “exaggerated” response is that the judgement often lies with the clinician, not the patient, relying on an objective notion of what a patient might be expected to feel, which conflicts with the entirely subjective nature of pain.^53^ Given evidence of implicit bias in clinicians’ judgments of the trustworthiness of patients’ pain reports—particularly bias against female patients—it is arguable that clinicians’ judgment should not determine whether a pain response is exaggerated or maladaptive versus “normal” or “appropriate.” Consistent with this perspective, a major theme emerging from the responses in this study was a concern that the term pain catastrophizing is stigmatizing, connoting dramatization, faking, or hysteria. The professionals indicated that this label may negatively influence healthcare providers’ judgments about these patients. Research indicates that negative expectations (or negative stereotypes) of patients significantly bias clinician judgment and patient care.^54^ For example, Zhang et al. (2023)^55^ conducted two experimental studies examining the impact of implicit gender bias on pain estimation and treatment decisions. They found that perceivers’ belief that women tend to exaggerate pain more than men was associated with lower pain estimations for women and judgments favoring psychotherapy over pain medicine. Future research should investigate whether clinicians’ knowledge of a patient’s pain catastrophizing score influences judgments of pain intensity or treatment decision-making.

Indeed, in the prior patient survey study, one-third of participants expressed problems with the term, associating it with invalidation of their pain experience and a lack of clinician empathy.^13^ It is important to distinguish criticism of the term from the construct itself. Most, if not all, clinicians agree on the importance of addressing harmful patterns of thinking about pain, such as believing one’s life is over due to pain. Addressing this kind of thinking (the construct) is clearly important. However, labeling the construct as “pain catastrophizing” (the term) may be perceived as demeaning to the person, and perhaps an inaccurate descriptor of the construct.^13,16,56^ This highlights a gap between clinicians’ accurate clinical understanding of pain catastrophizing and the compassionate and effective use of the term in practice. Given its widespread use, clinicians should consider the term’s impact on patient trust and perceived clinician empathy while exploring ways to enhance patient receptivity to effective pain management treatments.

Ultimately, the current data illustrate variations in clinician and researcher perceptions and interpretations of pain catastrophizing as a term and concept. A useful distinction might be between brief episodes and persistent severe patterns of pain catastrophizing (reflected in high scores on catastrophizing measures), for which treatment might be beneficial. Developing patient-centered terminology, informed by stakeholders with lived experience, is acknowledged as a priority. Finally, the use of pain catastrophizing in media stories falls outside of careful clinician messaging, underscoring the need for intentional labeling of the construct. While consensus is lacking, some patients^13^ and clinicians view the term as stigmatizing, suggesting a renaming may be required.

Patient experiences of pain-related stigma extends beyond the term pain catastrophizing and its use or misuse. This study was part of a broader patient-centered and patient-led effort involving multiple patient stakeholders, including leaders of national and international patient organizations, who reported stigma associated with the term in clinical care and media stories, both for themselves and their constituents. The research was a response to patient requests to investigate the term’s impacts, as many felt it uniquely ascribed blame to patients.

Psychologists generally receive extensive training on patient-client communications, sensitivities, and potential stigma; however, psychologists comprised only 15% of the current sample. Moreover, roughly 35% of the sample did not identify as pain specialists and may be less aware of the stigmas experienced by patients with chronic pain. Additionally, while this research represents an initial exploration of clinician and research perspectives, we acknowledge that any potential change in nomenclature would require additional rigorous studies to support such a goal.

Further research is needed to establish effective educational interventions and training to improve clinician communication. However, the greatest capacity for improvements may lie in clinicians’ ability to deliver messages and treatments that validate patients and enhance a sense of safety. Future studies may also examine how stigma associated with the term impacts individuals’ willingness to engage in pain psychology services or other needed care. Promoting respectful, sensitive clinician-patient communication can enhance patient receptivity to treatments and improve pain management and coping.

### 4.10. Strengths and limitations

Most participants in our study were US-based, female, English-speaking clinicians with >10 years of clinical experience, limiting generalizability beyond these groups.

Clinician discipline, pain specialty designation, and duration of practice data were lacking in this study and are noted to be important factors for future research; the current study provides only a broad understanding of clinicians’ experiences, term usage, and perspectives.

Using online surveys for data collection has strengths and limitations. Surveys are cost-effective and efficient, allowing for greater reach and potential response rates.^13,45,57,58^ However, they risk bias from selective participation^59^ and subjective responses, which may include fraudulent inputs^45^ that could impact accuracy and generalizability.^57^

The study name and survey language could have introduced negative bias into participant responses; the study website, called “renamepc. stanford.edu,” was named based on requests from multiple patient stakeholders.^13^ The introductory language on the website acknowledged our aim to understand whether a replacement term was needed, and the survey itself introduced the term as potentially unhelpful and distressing,^13^ again, guided by patient requests. Finally, due to the size of the dataset and large number of codes generated, it was not practical to conduct or report a granular examination of patterns by participant characteristics (e.g., professional discipline, pain specialization, or experience). Moreover, the analysis relied on only a few free responses, capturing many participant perspectives but limiting the richness of the responses provided. Future research on this topic would benefit from adopting interview or focus group methods to gain deeper insights from participants.

Despite these limitations, this study has several strengths. First, the study included perspectives from a large international sample, making it, to our knowledge, the largest study on chronic pain terminology among international clinicians and researchers. The multi -stakeholder design of the study included 12 patient stakeholders from 4 countries, 38 scientific collaborators from 9 countries, 7 national patient organizations, and 2 international patient advocacy organizations.^13^

The coding framework for the study was inductive, informed by data. Due to similarity in the items used in the prior study, a priori knowledge informed an initial structuring of the data. However, the analytic process was inductive, iterative, and did not track with the prior (patient) study. Multiple iterations by coders with varying levels of knowledge on chronic pain terminology enhanced the reliability and generalizability of the qualitative results.

This content analysis provides a diverse foundation of professional opinions surrounding the term and its usage, offering opportunities to align patient and professional priorities for its application in pain treatment. These results may guide future research and clinical efforts to improve patient-clinician communication and patients’ clinical experiences. Key areas for intervention include education on effective clinical communication that validates patient experiences and empowers them in their treatment decisions. Future research should also focus on consistency in knowledge and approaches across professional disciplines and investigate whether patient-informed education of clinicians on chronic pain experiences improves clinical encounters.

### 4.11. Disclosures

Dr. Darnall receives royalties for four pain treatment books she has authored or coauthored. She is the principal investigator for two pain research awards from the Patient-Centered Outcomes Research Institute. Dr. Darnall is principal investigator for two NIH grants. Dr. Darnall serves on the Board of Directors for the American Academy of Pain Medicine, is on the Board of Directors for the Institute for Brain Potential, and is on the Medical Advisory Board for the Facial Pain Association. Dr. Darnall is a scientific member of the NIH Interagency Pain Research Coordinating Committee, a former member of the Centers for Disease Control and Prevention Opioid Workgroup (2020–2021), and a current member of the Pain Advisory Group of the American Psychological Association. Dr. Mackey receives research funding from the NIH, Food and Drug Administration, and Patient-Centered Outcomes Research Institute (administered through Stanford University). He is an unpaid advisor to both ACTTION (Analgesic, Anesthetic, and Addiction Clinical Trial Translations, Innovations, Opportunities, and Networks) on their oversight committee, and the American Chronic Pain Association (ACPA) and the Chronic Pain Research Alliance for their scientific oversight. Geert Crombez receives research funding from FWO-Flanders, Versus Arthritis UKRI (Advanced Pain Discovery Platform PAINSTORM), Stand Up against Cancer, and Ghent University. He is consultant for MoveUp regarding the implementation of behavioural change techniques in digital interventions. The authors declare that the research was conducted in the absence of any commercial or financial relationships that could be construed as a potential conflict of interest. This effort was supported in part by grants from the National Institutes of Health NIDA K24 DA053564 (BDD) and NIA R01 AG063241 (LMM).

## Supplementary Material

MMC1

## Figures and Tables

**Table 1:** Sample characteristics by profession.

Full Sample (Clinician and Researcher; n=1397)			
Characteristic	Response	n (%)	
Professional category	Clinician	1098 (78.6)	
	Researcher	284 (20.3)	
	Missing	15 (1.2)	
Age M, (SD)		56.67 (4.04)	
Gender	Female	856 (61.3)	
	Male	482 (34.5)	
	Non-Binary	12 (0.9)	
	Prefer not to say	21 (1.5)	
	Missing	26 (1.8)	
Clinicians Only (n=1098)	Characteristic	Response	n (%)
	Pain specialist	A pain specialist	698 (63.6)
		Non-pain specialist	391 (35.6)
		Missing	9 (0.8)
	Duration of time in clinical practice	Less than 1 year	68 (6.2)
		1–4 years	173 (15.8)
		5–10 years	215 (19.6)
		More than 10 years	641 (58.4)
	Clinical discipline	Physical Therapist	361 (32.9)
		Physician	285 (26.0)
		Psychologist	162 (14.8)
		Nurse	114 (10.4)
		Other*	131 (12.0)
		Missing	45 (4.1)

* The other category includes Trainee or Students (n=24), Social workers (n=19), Massage Therapists (n=17), Physician Assistants (n=14), Medical Assistants (n=5), Acupuncturists (n=3) and other professions.

**Table 2:** Frequency of healthcare professionals responses to open-ended survey items.

Survey items	Participants, n (%)
Please briefly describe what it [pain catastrophizing] means to you. If you are unsure, please just jot down a few words that come to mind that you associate with the term.	1102 (78.9)
Clinicians only: If yes, (you use the term with patients) can you describe how patients respond to the term?	*312 (84.5)
Please tell us what first comes to your mind when you hear the term pain catastrophizing?	1130 (80.9)
Please feel free to add any further comments or thoughts about what would be a better term for pain catastrophizing?	704 (50.4)
Is there anything else you would like to tell us on this topic? Please feel free to attach a separate page if you’d like to provide further comments or insights.	548 (39.3)

* clinician participants who responded yes to using the term with patients (n=369).

## Data Availability

Written data requests should be sent to the corresponding author for review, approval and access.
