# Supplementary material for: Clinician and researcher responses to the term pain catastrophizing and whether new terminology is needed: Content analysis of international, cross-sectional, qualitative survey data
Source: J Pain. Author manuscript; Available in PMC 2025 Apr 1. (PMC11929406; doi:10.1016/j.jpain.2025.105330)
Supplement: MMC1 [file NIHMS2058287-supplement-MMC1.docx]

**Time for a Change?**

**Healthcare Provider and Pain Researcher Perceptions of the Term ‘Pain Catastrophizing’**

OUR GOAL:   With this survey, we aim to understand healthcare provider and pain researcher perspectives on the term “**pain catastrophizing**.”  This research is motivated by reports from patients and providers alike that the term ‘pain catastrophizing’ is unhelpful and may contribute to patient alienation and distress. As such, we are distributing a patient survey internationally to learn about the patient perspective. Our larger goal in this patient-centered work is to explore new terminology that is broadly acceptable to *all stakeholders*, and promotes good patient engagement and collaboration with healthcare providers to achieve best outcomes for their pain treatment.

We appreciate your participation and opinions, and thank you for your thoughtful contributions. All answers are anonymous and will be kept confidential.

1. Do you consider yourself *primarily* a:
   - 1. Healthcare provider
     2. Researcher

If they endorse (b) researcher skip down to 5.

Only if they endorse (a) healthcare provider

- Do you consider yourself:
  1. a pain specialist
  2. non-pain specialist

3. For how long have you been in clinical practice treating patients?

A.  Less than 1 year

B.  1-4 years

C.  5-10 years

D.  More than 10 years

1. What is your professional discipline?           [drop down]

- physical therapist
- psychologist
- social worker
- occupational therapist
- physician
- nurse
- physician assistant
- medical assistant
- mental health therapist
- massage therapist
- acupuncturist
- surgeon
- trainee / student
- Other ________________________________________________________

5. What is your age? __________   [drop down, numeric age, not DOB]

6. What is your gender?  __________  [drop down]

7. In which country do you reside? ________________________  [drop down]

8. Have you heard of the term ‘pain catastrophizing’?

A. Yes

B. No

1. Please briefly describe what it means to you. If you are unsure, please just jot down a few words that come to mind that you associate with the term.

(blue questions only for healthcare providers)

1. Have you ever used the term ‘pain catastrophizing’ in the context of your communications with patients?

Yes/No

1. [branching logic] If yes, can you describe how patients respond to the term?

1. [branching logic from #4] How frequently do your patients react negatively to your use of the term pain catastrophizing?

- Never
- Rarely
- Occasionally
- About half the time
- Often
- Almost always
- Every time

comments:  ______________________________________

1. Please rate how important you feel it is to create a new term for pain catastrophizing, one that would be acceptable to most patients, providers, and researchers?

- Not at all important
- Minor importance
- Somewhat important
- Neutral
- Important
- Great importance
- Greatest importance

**This is the definition of pain catastrophizing**:  Pain catastrophizing refers to how we respond to pain we have right now, or to pain we *expect* to have in the future. It includes thoughts we may have about pain (e.g., “*I can’t stop thinking about how much it hurts*.”), feelings about pain (such as helplessness) and expectations for future pain (e.g., “I worry that my pain will only get worse.”).

While the degree of pain catastrophizing and level of pain intensity we experience are related, research shows that they are different. We can control for pain intensity in research studies (by keeping  it constant) and see that pain catastrophizing – *our level of pain-specific distress* -- changes how pain is processed in the central nervous system.

13. Please tell us what first comes to your mind when you hear the term pain catastrophizing?

It is important to study how the mind (our beliefs, thoughts and emotions) relates to what we feel in the body. To do this, pain professionals and researchers use labels that make sense, communicate ideas, describe experiences that are relevant to patients with pain. **Pain catastrophizing** is one of those terms, but use of this term has created problems for some patients and healthcare providers.

14. Below are a few initial possibilities for a new term for **pain catastrophizing**. Please circle the number below each term to indicate your approval/disapproval rating for each proposed new term.

**Pain-specific distress**

   1       2   3   4   5     6     7

Strongly Disapprove   Mostly Neutral Mostly Approve Strongly

Disapprove Disapprove Approve Approve

**Negative pain mindset**

      1     2     3   4     5     6     7

Strongly Disapprove   Mostly Neutral Mostly Approve Strongly

Disapprove Disapprove Approve Approve

**Negative pain appraisal**

      1     2     3   4     5     6     7

Strongly Disapprove   Mostly Neutral Mostly Approve Strongly

Disapprove Disapprove Approve Approve

**Pain-related worry**

      1     2     3   4     5     6     7

Strongly Disapprove   Mostly Neutral Mostly Approve Strongly

Disapprove Disapprove Approve Approve

**Pain-related anxiety**

      1     2     3   4     5     6     7

Strongly Disapprove   Mostly Neutral Mostly Approve Strongly

Disapprove Disapprove Approve Approve

**Pain-related concerns**

      1     2     3   4     5     6     7

Strongly Disapprove   Mostly Neutral Mostly Approve Strongly

Disapprove Disapprove Approve Approve

**Pain-related fear**

      1     2     3   4     5     6     7

Strongly Disapprove   Mostly Neutral Mostly Approve Strongly

Disapprove Disapprove Approve Approve

**Pain-related distress**

      1     2     3   4     5     6     7

Strongly Disapprove   Mostly Neutral Mostly Approve Strongly

Disapprove Disapprove Approve Approve

**Pain Vigilance**

      1     2     3   4     5     6     7

Strongly Disapprove   Mostly Neutral Mostly Approve Strongly

Disapprove Disapprove Approve Approve

15. Please feel free to add any further comments or thoughts about what would be **a better term** for **pain catastrophizing**?  (There are no right or wrong answers, or bad ideas.)

16.  Is there anything else you would like to tell us on this topic? Please feel free to attach a separate page if you’d like to provide further comments or insights.

____________________________________________________________________________________

We will be collecting many suggestions from patients and healthcare providers for a new and better term that is broadly acceptable. We will collate survey responses and distribute a final survey in the near future using similar methods. **Please join our mailing list if you wish to receive our final survey by direct email.**

Thank you so much for sharing your thoughts and experiences with us. We hope to use all the valuable information you have shared to bring more helpful and compassionate terminology to the practice of communicating about the experience and treatment of pain.
